# Transcriptome Analysis Reveals the Molecular Mechanisms by Which *ADAMTS1* Influences the Proliferation of Ovarian Granulosa Cells in Sheep

**DOI:** 10.3390/ani15162354

**Published:** 2025-08-11

**Authors:** Rongqing Li, Wenjia Zhang, Yuanshuai Gao, Zhiqiang Xie, Jiangfeng He, Qinyuan Fang, Mohamed El-Sherbiny, Min Gao, Zheng Wang, Teng Zhang, Fang Liu, Biao Wang, Yongbin Liu

**Affiliations:** 1 Key Laboratory of Herbivorous Livestock Reproductive Regulation, National Sheep Genetic Evaluation Center, Inner Mongolia University, Hohhot 010030, China; rong-qing.li@mail.imu.edu.cn (R.L.); zhang1552024@163.com (W.Z.); gys990928@163.com (Y.G.); 15066545108@163.com (Z.X.); fangqinyuan@163.com (Q.F.); min.gao@imu.edu.cn (M.G.); wangzheng@imu.edu.cn (Z.W.); zhangteng428@163.com (T.Z.); 2 Inner Mongolia Academy of Agricultural & Animal Husbandry Sciences, Hohhot 010031, China; 13704781032@163.com (J.H.); liuf185@163.com (F.L.); 3 Department of Dairy Science, National Research Centre, 33 Bohouth St., Dokki, Giza 12622, Egypt; elsherbiny.nrc.eg@gmail.com; 4 Department of Animal Genetics, Breeding, and Reproduction, College of Animal Science, Inner Mongolia Agricultural University, Hohhot 010070, China

**Keywords:** *ADAMTS1*, *PSAT1*, *SLC6A9*, granulosa cells, proliferation, apoptosis, follicle, follicular development, follicular atresia, oocyte

## Abstract

Normal proliferation of ovarian granulosa cells is essential for follicular development. The results of this study showed that ADAMTS1 was primarily localized in the cytoplasm of granulosa cells in sheep ovarian follicles, as revealed by immunohistochemistry and immunofluorescence staining. Knockdown and overexpression experiments of *ADAMTS1* in granulosa cells demonstrated that the number of EdU-positive cells significantly decreased in the knockdown group (*p* < 0.05), while the expression levels of *Bax* (*p* < 0.05), *Bax*/*Bcl2* (*p* < 0.01), and *caspase3* (*p* < 0.05) were significantly upregulated, indicating that knockdown of *ADAMTS1* markedly inhibited granulosa cell proliferation. In contrast, overexpression of *ADAMTS1* significantly promoted cell proliferation. Transcriptome sequencing revealed that *PSAT1* and *SLC6A9* were significantly downregulated in the knockdown group and significantly upregulated in the overexpression group, which was confirmed by Quantitative Polymerase Chain Reaction (Q-PCR) (*p* < 0.05). KEGG enrichment analysis showed that *PSAT1* was significantly enriched in the glycine, serine and threonine metabolism and vitamin B6 metabolism pathways. Molecular docking analysis indicated a stable binding interface between ADAMTS1 and PSAT1. Based on these findings, we speculate that *ADAMTS1* may regulate amino acid metabolism in ovarian granulosa cells by modulating the expression of SLC6A9, which in turn affects *PSAT1* in the glycine, serine, and threonine metabolism and vitamin B6 metabolism pathways, thereby influencing granulosa cell proliferation.

## Simple Summary

The normal proliferation of ovarian granulosa cells (GCs) is crucial for follicular development and female reproductive capacity. ADAM metallopeptidase with thrombospondin type 1 motif 1 (*ADAMTS1*) is a member of the metalloproteinase family. Current research on *ADAMTS1* mainly focuses on follicle rupture, oocyte release, and the morphogenesis of the cumulus-oocyte complex (COC) matrix. However, the mechanism by which *ADAMTS1* affects granulosa cell proliferation remains unclear. Therefore, this study aims to explore the molecular mechanism by which *ADAMTS1* regulates the proliferation of ovine ovarian granulosa cells. We found that knockdown of *ADAMTS1* significantly inhibited the proliferation of granulosa cells, while overexpression of *ADAMTS1* significantly promoted their proliferation. Phosphoserine aminotransferase 1 (*PSAT1*) and Solute carrier family 6 member 9 (*SLC6A9*) were significantly downregulated in the knockdown group and significantly upregulated in the overexpression group. We discovered a stable binding interface between ADAMTS1 and PSAT1. We speculate that *ADAMTS1* may regulate amino acid metabolism in ovarian granulosa cells by modulating the expression of *SLC6A9*, which in turn affects *PSAT1* in the glycine, serine, and threonine metabolism and vitamin B6 metabolism pathways, thereby influencing granulosa cell proliferation.

## 1. Introduction

The ovary is the central reproductive organ in female mammals, and the follicle is the basic functional unit of the ovary [1]. Follicular development is influenced by the extracellular matrix (ECM) and the ovarian stromal microenvironment, and is also tightly regulated by the health status of oocytes and granulosa cells [2]. During follicular development, gap junctions form between granulosa cells and oocytes [3]. Through paracrine signaling, oocytes influence gene expression involved in glycolytic activity and regulate amino acid uptake in granulosa cells, thereby modulating their metabolic processes [1]. Granulosa cells not only serve as nutritional support cells but also function as secretory cells, supplying oocytes with amino acids, glucose, and other substances, thus promoting the development of both oocytes and follicles [4,5]. However, most follicles undergo atresia during development, leading to ovarian dysfunction, and granulosa cell apoptosis is the primary cause of follicular atresia [6]. Therefore, the proper proliferation of granulosa cells is crucial for follicular development and female reproductive capacity [7]. Elucidating the key molecular mechanisms that regulate the proliferation of ovarian granulosa cells is of great significance for understanding follicular development.

Follicular development is regulated not only by the health status of oocytes and granulosa cells but also by the extracellular matrix (ECM) and the ovarian stromal microenvironment. ADAMTS1 is a member of the ECM-associated metalloproteinase family and plays a critical role in female reproduction [8]. ADAMTS1 has been identified in the ovaries of humans, mice, cattle, horses, and pigs, and is primarily produced by follicular granulosa cells [9]. In *ADAMTS1*-deficient mice, abnormal ovarian morphology, significantly reduced ovulation numbers, follicular atresia, and decreased fertilization rates have been observed [8,10], indicating the essential role of *ADAMTS1* in follicular development. In bovine preovulatory follicles, the mRNA abundance of *ADAMTS1* in granulosa cells is significantly higher during the ovulatory phase than in preovulatory follicles, further suggesting its importance in ovulation [9]. To date, studies on *ADAMTS1* in mammalian ovaries have mainly focused on ovulation and fertilization. The proteolytic activity of *ADAMTS1* is known to mediate the morphogenesis of the follicular wall and the cumulus-oocyte complex (COC) matrix, as well as the subsequent degradation of versican, which is required after fertilization [11]. However, current studies on *ADAMTS1* in sheep have largely focused on fertility. Litter size in sheep is a trait controlled by multiple genes, and *ADAMTS1* has been identified as one of the key genes influencing reproductive performance [12]. SNP polymorphisms of *ADAMTS1* are involved in regulating litter size in Hu sheep and Small Tail Han sheep [13], and similar associations between *ADAMTS1* polymorphisms and litter size have been found in goats [14]. Since high fecundity is associated with the production of a large number of mature follicles [15], it is crucial to investigate the expression pattern and regulatory mechanism of *ADAMTS1* in sheep follicles. However, little is known about how *ADAMTS1* regulates follicular development and granulosa cell proliferation in sheep.

In the gap junctions between follicular granulosa cells and oocytes, granulosa cell-regulated amino acid metabolism is crucial for follicular development, and the glycine, serine, and threonine metabolism pathway represents a central network in amino acid metabolism. PSAT1 is a key rate-limiting enzyme in this pathway [16]. Studies have shown that *PSAT1* is involved in regulating cell proliferation in vitro. Overexpression of *PSAT1* in human U937 cells significantly promotes cell proliferation [17], whereas knockdown of *PSAT1* markedly inhibits the clonogenicity and cell cycle of ovarian cancer cells and promotes their apoptosis [16]. In patients with polycystic ovary syndrome, *PSAT1* is differentially expressed in granulosa cells corresponding to different nuclear maturation stages of oocytes, suggesting its role in regulating oocyte maturation and embryo quality [18]. The serine-glycine biosynthesis pathway involving *PSAT1* contributes to the production of folate, methionine, and vitamin B6. Vitamin B6 is an essential enzymatic cofactor required for biochemical reactions, involved in both the biosynthesis and degradation of amino acids [19], and plays a central role in amino acid metabolism [20]. As an aminotransferase, the catalytic activity of PSAT1 is highly dependent on its cofactor. In the human brain, the biosynthesis of serine relies on vitamin B6 [21]. Therefore, the metabolic status of vitamin B6 may influence PSAT1 enzyme activity and the flux of serine synthesis. In current research, *PSAT1* has been identified as an oncogene associated with metastasis and poor prognosis in various malignancies, including lung cancer, liver cancer, colorectal cancer, breast cancer, ovarian cancer, and endometrial cancer [16,22,23]. However, studies on *PSAT1* in follicular development, particularly in sheep and their ovaries, remain limited.

Based on the potential signaling pathways and microenvironment regulated by *ADAMTS1*, as well as the central role of *PSAT1* and its related metabolic pathways in cell proliferation, we hypothesize that *ADAMTS1* may regulate the proliferation of ovine ovarian granulosa cells by modulating the expression of *PSAT1* in the glycine, serine, and threonine metabolism and vitamin B6 metabolism pathways. We propose a novel regulatory axis—*ADAMTS1*–*PSAT1*–metabolic pathway—which may play a key role in follicular development and granulosa cell function. Therefore, this study aims to investigate the regulatory relationship between *ADAMTS1* and *PSAT1* in sheep ovaries and to explore the mechanism by which *ADAMTS1* affects granulosa cell proliferation. We hope to provide a preliminary theoretical basis for reproductive research in sheep.

## 2. Materials and Methods

### 2.1. Animal and Specimen Collection

Ninety healthy 18-month-old Mongolian sheep (Sunite sheep), all of which had lambed once and exhibited estrus behavior prior to sampling, were selected for this study. Sample collection was conducted in October, with an outdoor temperature of 18 °C and humidity of 37% on the day of collection. A total of 180 ovarian tissue samples were surgically collected from healthy Mongolian sheep at a slaughterhouse in Siziwang Banner, Ulanqab City, Inner Mongolia Autonomous Region. All animal-related procedures were reviewed and approved by the Institutional Animal Care and Use Committee of Inner Mongolia University (Hohhot, Inner Mongolia, China), under approval number 2024/005. The study was conducted in strict accordance with the Administrative Regulations on Laboratory Animals. Comprehensive measures were implemented to minimize animal distress, and all procedures adhered to established animal welfare and ethical standards.

### 2.2. Hematoxylin–Eosin Staining

Fresh ovarian tissues were immersed in 4% fixative solution (Solarbio, Beijing, China) and preserved for 24 h at room temperature. Following fixation, samples underwent a standard dehydration protocol, paraffin infiltration, and embedding. Paraffin blocks were sectioned, and the resulting tissue slices were deparaffinized and subjected to hematoxylin staining (Servicebio, Wuhan, China) followed by eosin (Servicebio, Wuhan, China) counterstaining. After dehydration and mounting, histological structures were visualized using a light microscope (Nikon, Tokyo, Japan).

### 2.3. Immunohistochemical Staining

Fresh ovarian tissues were fixed in a designated fixative for 24 h, followed by standard dehydration, paraffin infiltration, and embedding procedures. Serial sections were then cut from the paraffin blocks, deparaffinized, and subjected to antigen retrieval. Endogenous peroxidase activity was quenched prior to blocking with serum (Servicebio, Wuhan, China). Sections were incubated overnight at 4 °C with a primary antibody targeting ADAMTS1 (Biorbyt, Beijing, China), followed by incubation with an appropriate secondary antibody (Kangwei, Taizhou, China). Immunostaining was developed using 3,3′-diaminobenzidine (DAB) (OriGene Technologies, Rockville, MD, USA) substrate, and nuclei were counterstained. Finally, after dehydration and coverslipping, the stained sections were examined microscopically.

### 2.4. Isolation and Culture of Granulosa Cells and Oocytes

Fresh ovarian tissues were thoroughly washed with physiological saline solution (Kelun, Sichuan, China). Follicles on the tissue surface were excised using sterile surgical blades, and follicular fluid was collected and allowed to settle by gravity. Under a stereomicroscope (Nikon, Tokyo, Japan), oocytes surrounded by granulosa cell layers exceeding five were selected. These oocytes were then transferred into an in vitro maturation medium supplemented with 10% fetal bovine serum (Gibco, Carlsbad, CA, USA) and incubated at 38.5 °C in an incubator (Thermo Fisher Scientific, Waltham, MA, USA). Granulosa cells were harvested and cultured in high-glucose Dulbecco’s Modified Eagle Medium (DMEM) containing 10% fetal bovine serum (VivaCell, Shanghai, China) at 37 °C in an incubator (Thermo Fisher Scientific, Waltham, MA, USA).

### 2.5. Immunofluorescence Staining

Granulosa cells were plated in 24-well plates and divided into three groups: the NC group (without primary antibody incubation), the FSHR group (incubated with FSHR primary antibody), and the ADAMTS1 group (incubated with ADAMTS1 primary antibody). Following 24 h culture, cells were fixed in 4% tissue fixative for 30 min, then permeabilized with 0.2% Triton X-100 (Coolaber, Beijing, China) for 20 min. Non-specific binding sites were blocked by treatment with 5% bovine serum albumin (BSA) (Servicebio, Wuhan, China) for 3 h. The cells were subsequently incubated overnight at 4 °C with primary antibodies targeting FSHR and ADAMTS1 (Bioss, Beijing, China). After washing, fluorescently labeled secondary antibodies (Bioss, Beijing, China) were applied for 2 h. Nuclear staining was performed using DAPI (Solarbio, Beijing, China) for 2 min. Fluorescence imaging was conducted using a fluorescence microscope (Nikon, Tokyo, Japan).

Granulosa cells were seeded into 24-well plates and cultured for 24 h. Subsequently, cells were fixed with 4% tissue fixative for 30 min, then permeabilized using 0.2% Triton X-100 (Coolaber, Beijing, China) for 20 min. Non-specific binding was blocked by incubation with 5% bovine serum albumin (BSA) (Servicebio, Wuhan, China) for 3 h. The cells were then incubated overnight at 4 °C with primary antibodies targeting FSHR and ADAMTS1 (Bioss, Beijing, China). After washing, cells were exposed to fluorescently labeled secondary antibodies (Bioss, Beijing, China) for 2 h. Nuclear counterstaining was performed using DAPI (Solarbio, Beijing, China) for 2 min. Fluorescence imaging was conducted using a fluorescence microscope.

### 2.6. Synthesis of siRNA

Utilizing the NCBI *ADAMTS1* gene identifier (Gene ID: 100415773), four siRNA sequences were designed through computational tools and subsequently synthesized by Suzhou Jima Gene Co., Ltd. (Jima, Suzhou, China) for gene silencing applications.

### 2.7. Construction of Overexpression Vectors

Primers targeting the *ADAMTS1* gene were designed utilizing Primer Premier 5.0 software (Table 1). The coding sequence (CDS) of *ADAMTS1* was amplified via polymerase chain reaction (PCR) (Thermo Fisher Scientific, Waltham, MA, USA) and the resulting amplicons were validated by agarose gel electrophoresis (Bio-Rad Laboratories, Hercules, CA, USA) followed by gel purification. The purified PCR fragments were subjected to double digestion using restriction enzymes EcoR I and BamH I (Vazyme, Nanjing, China). Digested products were analyzed by agarose gel electrophoresis and purified. Concurrently, the pcDNA3.1 expression vector (TIANGEN, Beijing, China) underwent restriction digestion with the same enzymes, and the linearized plasmid was verified and isolated by gel electrophoresis. The *ADAMTS1* insert was then ligated into the prepared vector backbone. The recombinant plasmid construct was transformed into DH5α chemically competent cells (TIANGEN, Beijing, China). Transformed colonies were selected on ampicillin-containing solid media. Plasmid DNA from positive clones was extracted (TIANGEN, Beijing, China), digested with EcoR I and BamH I to confirm correct insertion via gel electrophoresis, and subsequently submitted to Shanghai Shenggong Bioengineering Co., Ltd. for sequencing validation.

### 2.8. Transfection of Granular Cells

Granulosa cells were seeded in 6-well plates and divided into five groups: si-NC (negative control), si-ADAMTS1-1 (knockdown), si-ADAMTS1-2 (knockdown), si-ADAMTS1-3 (knockdown), and si-ADAMTS1-4 (knockdown). Cells were transfected with siRNA when reaching approximately 70% confluence. OPTI-MEM medium (Gibco, Carlsbad, CA, USA) was gently combined with GP-transfect-Mate reagent (GenePharma, Shanghai, China) and incubated at room temperature for 5 min. Separately, OPTI-MEM was mixed with the siRNA solution and incubated for an additional 5 min. These two solutions were then gently combined and left to complex for 20 min at room temperature. The resulting transfection complexes were slowly added to the cells, which were subsequently maintained in a humidified incubator at 37 °C with 5% CO_2_.

Granulosa cells were plated in 6-well plates and divided into two groups: vector ctrl (empty vector control) and ADAMTS1-OE (*ADAMTS1* overexpression). Granulosa cells were seeded into six-well plates and allowed to reach approximately 70% confluence before transfection. OPTI-MEM medium (Gibco, Carlsbad, CA, USA) was gently combined with GP-transfect-Mate reagent (GenePharma, Shanghai, China) and incubated at room temperature for 5 min. Separately, OPTI-MEM was mixed with the siRNA solution and incubated for an additional 5 min. These two solutions were then carefully combined and incubated for 20 min to form transfection complexes. The mixture was then slowly added to the cells, which were subsequently cultured under standard conditions at 37 °C with 5% CO_2_.

### 2.9. RNA Extraction and RT-qPCR

Following transfection with si-NC, si-ADAMTS1, vector ctrl, or ADAMTS1-OE, total RNA was isolated using TRIzol reagent (TIANGEN, Beijing, China) and purified according to standard procedures. Genomic DNA contamination was eliminated, and first-strand cDNA synthesis was performed using a two-step reverse transcription protocol (Takara, Beijing, China). The RT-qPCR reaction mixture (20 μL total volume) consisted of 10 μL cDNA template, 1 μL PrimeScript RT Enzyme Mix I, 1 μL RT Primer Mix, 4 μL5 × PrimeScript Buffer 2, and 4 μL RNase-free water (TIANGEN, Beijing, China). GAPDH was employed as the endogenous control gene. Primer details are provided in Table 2. Relative quantification of gene expression was conducted utilizing the 2^−ΔΔCt^ method.

### 2.10. Western Blotting Analysis

After 48 h of transfection with si-NC, si-ADAMTS1, vector ctrl, or ADAMTS1-OE, adherent cells were harvested by gentle scraping with sterile polystyrene scrapers (Corning, Harrodsburg, KY, USA) to preserve membrane integrity. Total protein was extracted using a protein extraction kit (Solarbio, Beijing, China), with lysis performed on ice for 30 min. Following centrifugation, the supernatant was mixed with 5× protein loading buffer (Bioss, Beijing, China) and denatured in a water bath. Protein concentrations were quantified using a bicinchoninic acid (BCA) assay (Kangwei, Dongtai, China), and absorbance was measured at 562 nm. The concentration of each sample was calculated based on a standard curve (y = 0.9227x + 0.1416, R^2^ = 0.9998).

Proteins were resolved by SDS-PAGE (Solarbio, Beijing, China) using a 10% separating gel and a 5% stacking gel in 1× electrophoresis buffer. Electrophoresis was performed at 90 V for 110 min. After separation, protein bands were transferred onto polyvinylidene difluoride (PVDF) membranes (Immobilon, Shanghai, China) using a semi-dry transfer system (Bio-Rad Laboratories, Hercules, CA, USA) at 20 V for 55 min. Membranes were then blocked for 2 h, followed by overnight incubation at 4 °C with primary antibodies diluted at 1:1000. After washing, membranes were incubated with secondary antibodies (1:1000) for 2 h. Signal detection was carried out using enhanced chemiluminescence (ECL) reagents (BioLab, Beijing, China), and band intensities were quantified using Image-Pro Plus 6.0 software.

### 2.11. EdU Detection

At 24 h post-transfection with si-NC, si-ADAMTS1, vector ctrl, or ADAMTS1-OE, cells were treated with EdU-containing medium (RiboBio, Guangzhou, China) and incubated at 37 °C with 5% CO_2_ for 3 h. Following incubation, cells were fixed with 4% paraformaldehyde at room temperature for 15 min. For flow cytometric analysis, cells were enzymatically dissociated using trypsin (Gibco, Carlsbad, CA, USA) and subsequently fixed with the same fixative. Permeabilization was carried out using 0.3% Triton X-100 for 10 min at room temperature. The cells were then treated with Azide 555 Click reaction solution and incubated for 30 min in the dark. Nuclear staining was performed using DAPI for 10 min, also protected from light. EdU incorporation was assessed by fluorescence microscopy and quantified using flow cytometry (Thermo Fisher Scientific, Waltham, MA, USA).

### 2.12. Extraction and Sequencing of Transcriptome Samples

This study included a total of 12 samples, categorized into four experimental groups: si-NC, si-ADAMTS1, vector control, and ADAMTS1-OE, with three biological replicates in each group. Total RNA was isolated from all granulosa cell samples using TRIzol reagent (Takara, Beijing, China) and subsequently submitted to LC-Bio Technology Co., Ltd. (LC-Bio Technology, Hangzhou, China) for high-throughput sequencing. RNA purity and concentration were determined using a NanoDrop microvolume spectrophotometer (Thermo Fisher Scientific, Waltham, MA, USA). RNA integrity was evaluated using an Agilent Fragment Analyzer System (5300/5400, Agilent Technologies, Santa Clara, CA, USA, M5311AA), and RNA Integrity Number (RIN) values were used to assess sample quality. Paired-end sequencing (PE150) was conducted on the Illumina NovaSeq™ 6000 platform at LC-Bio Technology to generate transcriptomic data.

### 2.13. Sequencing Data Analysis

Sequence and filtering of Clean Reads: after obtaining the raw sequencing data, preprocessing and filtering were performed using Cutadapt (https://cutadapt.readthedocs.io/en/stable/, version: cutadapt-1.9, accessed on 20 March 2025) to remove reads containing adapter sequences, reads with excessively long poly-A or poly-G tails, reads with more than 5% unknown nucleotides (N), and low-quality reads with a base quality score below 20, thereby generating high-quality clean data. Sequence quality was assessed using FastQC (https://www.bioinformatics.babraham.ac.uk/projects/fastqc/, version: 0.11.9, accessed on 21 March 2025), which provided metrics, including Q20, Q30, and GC content of the clean data.

Alignment with Reference Genome: clean reads from each sample were aligned to the sheep reference genome (ARS-UI_Ramb_v3.0) using HISAT2 (https://daehwankimlab.github.io/hisat2/, version: hisat2-2.2.1, accessed on 21 March 2025).

Quantification of Gene Abundance: the aligned reads were assembled for each sample using StringTie (http://ccb.jhu.edu/software/stringtie/, version: stringtie-2.1.6, accessed on 24 March 2025) with default parameters. Subsequently, the transcripts from all samples were merged, and a comprehensive transcriptome was reconstructed using gffcompare (http://ccb.jhu.edu/software/stringtie/gffcompare.shtml, version: gffcompare-0.9.8, accessed on 24 March 2025). After generating the final transcriptome, transcript expression levels were estimated using StringTie and Ballgown (http://www.bioconductor.org/packages/release/bioc/html/ballgown.html, accessed on 24 March 2025) based on FPKM (Fragments Per Kilobase of transcript per Million mapped reads), and mRNA expression abundance was evaluated by calculating FPKM values.

Statistical Analysis: differential expression analysis between si-NC and si-ADAMTS1 groups, as well as between the vector control and ADAMTS1-OE groups, was conducted using DESeq2 (v 1.36.0). The resulting gene sets were subjected to multiple hypothesis testing correction using the Benjamini-Hochberg method to control the False Discovery Rate (FDR). Genes with a fold change ≥ 2 or ≤0.5 (|log2FC| ≥ 1) and a *q*-value < 0.05 (|log2FC| ≥ 1 & *q* < 0.05) were considered differentially expressed genes (DEGs) after BH-adjusted *p*-value correction.

Differentially expressed genes (DEGs) Analysis: correlation analysis was performed using R software (R 4.5.0), and principal component analysis (PCA) was conducted using the princomp function in R (http://www.r-project.org/, accessed on 25 March 2025). Functional enrichment analysis of DEGs was performed using Gene Ontology (GO) terms and Kyoto Encyclopedia of Genes and Genomes (KEGG) pathways. All DEGs were mapped to GO terms in the Gene Ontology database (http://www.geneontology.org/, accessed on 25 March 2025), and the number of genes associated with each term was calculated. A hypergeometric test was used to identify GO terms significantly enriched among the DEGs compared to the genomic background, with *p* < 0.05 considered statistically significant. Similarly, KEGG pathways with *p* < 0.05 were considered significantly enriched among DEGs.

### 2.14. Molecular Docking Analysis

The crystal structures of the ADAMTS1 and PSAT1 proteins were obtained from the PDB database (https://www.rcsb.org/, accessed on 25 March 2025). AutoDock Tools software (v 1.5.6) was used to preprocess the protein receptors and ligands, including the completion of missing amino acid residues, addition of hydrogen atoms, removal of small-molecule ligands and irrelevant ions, as well as energy minimization. AutoDock Vina software (v 1.2.5) was then used to perform molecular docking between the ligand and the receptor proteins. The possible positions, orientations, and conformations of the ligand at the active site were obtained, and the interaction energy between the ligand and receptor proteins was calculated to predict the most probable binding mode. PyMOL software (v 3.1.3) was used to visualize the predicted results.

### 2.15. Statistical Analysis

All experimental procedures were performed in triplicate biological replicates, with quantitative results expressed as mean ± SEM. GraphPad Prism 9.0 software was used for data analysis, and the differences were assessed using *t*-test or one-way ANOVA analyses. Statistical significance was established at *p* < 0.05, with significance levels denoted by asterisks (* for *p* < 0.05; ** for *p* < 0.01).

## 3. Results

### 3.1. ADAMTS1 Is Primarily Localized in the Follicular Granulosa Cells of the Ovine Ovary

**Figure 1:**
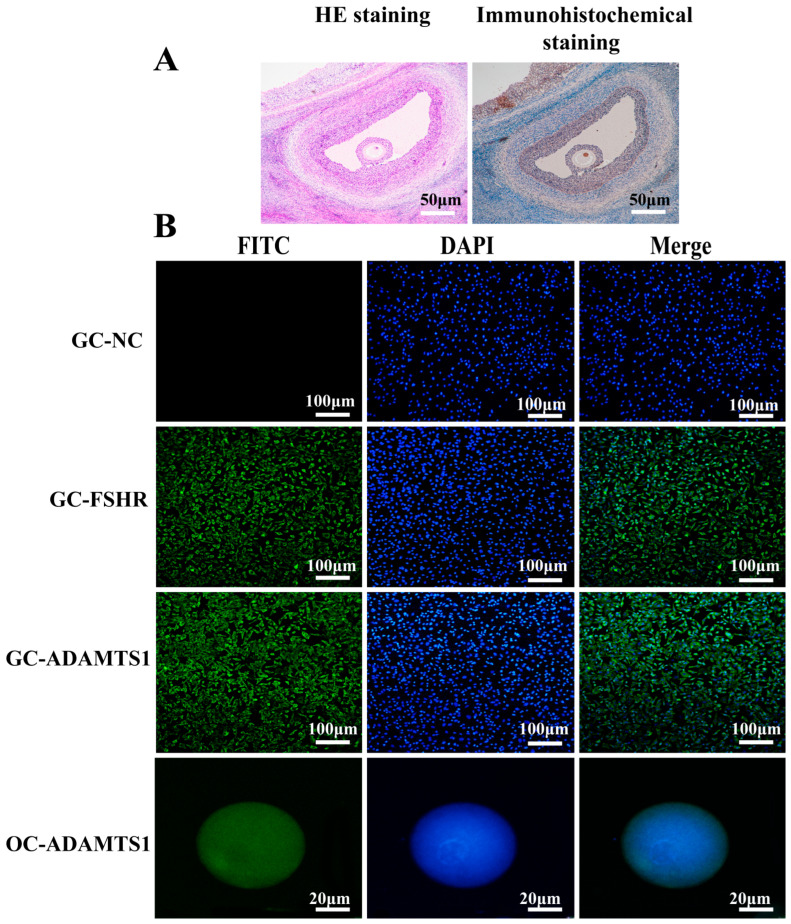
Predominant localization of ADAMTS1 in follicular granulosa cells of the ovine ovary. (**A**) Hematoxylin and eosin (HE) staining alongside immunohistochemical detection of ADAMTS1 in ovarian tissue. The left panel illustrates HE staining, while the right panel displays ADAMTS1 immunostaining (bar = 50 μm). (**B**) Immunofluorescence analysis of granulosa cells and oocytes. GC-NC: granulosa cells with negative control staining; GC-FSHR: granulosa cells immunostained for FSHR; GC-ADAMTS1: granulosa cells immunostained for ADAMTS1; OC-ADAMTS1: oocytes immunostained for ADAMTS1 (bar = 100 μm).

To elucidate the impact of *ADAMTS1* on ovarian cells, we initially investigated its precise localization within the ovine ovary. Ovarian tissues were obtained surgically from sheep and subjected to hematoxylin and eosin (HE) staining alongside immunohistochemical analysis targeting ADAMTS1. The immunostaining results revealed an absence of *ADAMTS1* expression in stromal cells, weak positivity in oocytes, and robust expression localized to the granulosa cells of ovarian follicles (Figure 1A). To further characterize *ADAMTS1* expression within follicular compartments, granulosa cells and oocytes were isolated from dissected follicles. Immunofluorescence staining was employed to assess *ADAMTS1* distribution. Follicle-Stimulating Hormone Receptor (FSHR), a definitive granulosa cell marker, was used to validate the identity of isolated cells. Strong *FSHR* immunoreactivity confirmed the successful isolation of granulosa cells. Notably, *ADAMTS1* exhibited weak nuclear expression in oocytes but strong cytoplasmic expression in granulosa cells (Figure 1B). These data collectively demonstrate that granulosa cells were effectively separated from ovine ovarian tissue and that ADAMTS1 predominantly localizes to the cytoplasm of follicular granulosa cells, implicating its potential role in follicular function.

### 3.2. Knockdown and Overexpression of ADAMTS1 in Ovarian Granulosa Cells

**Figure 2:**
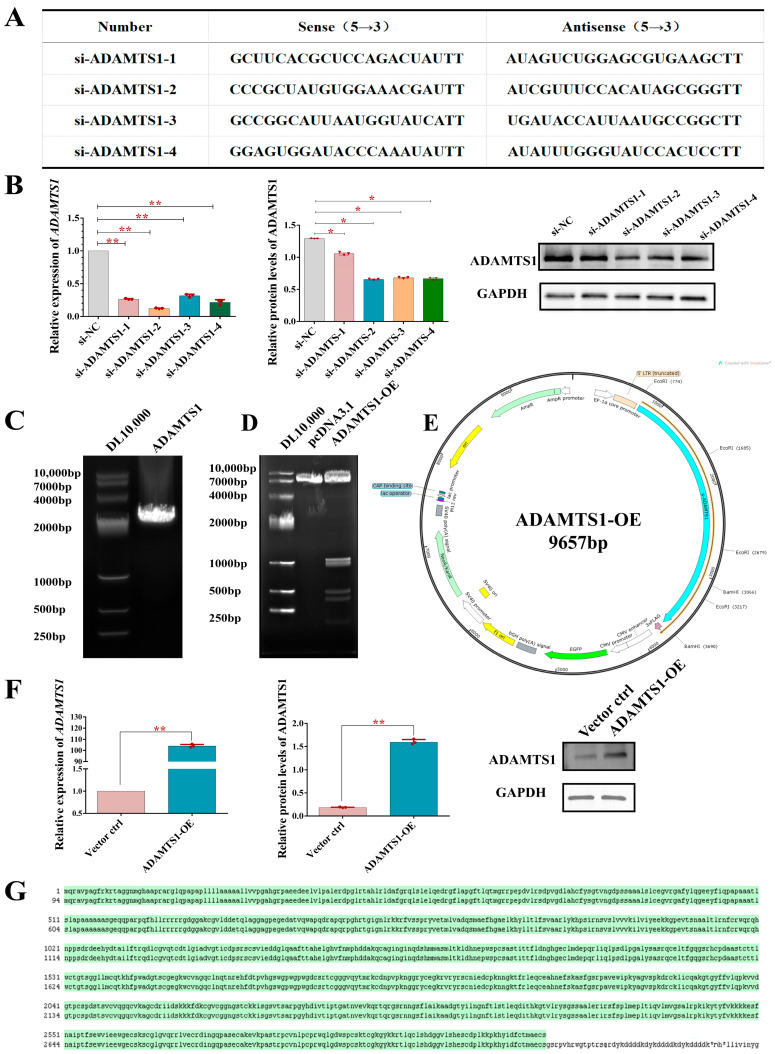
Design and validation of *ADAMTS1* knockdown and overexpression systems in ovarian granulosa cells. (**A**) Four siRNA targeting sequences against *ADAMTS1* were designed using an online prediction tool. (**B**) Knockdown efficiency was verified by quantifying *ADAMTS1* mRNA and protein levels post-transfection in granulosa cells. Among the four siRNA constructs tested, si-ADAMTS1-2 demonstrated the most robust knockdown efficiency, significantly reducing both *ADAMTS1* mRNA and protein expression levels in ovarian granulosa cells. (**C**) Agarose gel electrophoresis confirming successful PCR amplification of the *ADAMTS1* coding sequence (CDS). Using the DL10,000 DNA marker, agarose gel electrophoresis confirmed successful amplification of the *ADAMTS1* coding sequence (CDS), with the observed band (2903 bp) migrating between 2000 and 4000 bp, consistent with the expected product size. (**D**) Restriction enzyme digestion analysis using EcoR I and BamH I to verify linearized pcDNA3.1-EGFP vector and EGFP-ADAMTS1 recombinant plasmid. Using the DL10000 DNA marker, the linearized pcDNA3.1-EGFP vector migrated as a single band between 7000 and 10,000 bp, consistent with its expected size of 6750 bp. For the EGFP-ADAMTS1 recombinant plasmid, digestion with EcoRI and BamHI yielded fragments of 6750 bp, 994 bp, 900 bp, 475 bp, 387 bp, and 151 bp, all of which matched their predicted sizes by electrophoretic mobility. (**E**) Schematic representation of the *ADAMTS1* overexpression vector construct. The size of the overexpression vector EGFP-ADAMTS1 is 9657 bp. (**F**) Overexpression efficacy was assessed by measuring *ADAMTS1* transcriptional and translational levels following plasmid transfection. (**G**) Sanger sequencing chromatogram confirming the accurate insertion of EGFP-ADAMTS1 in the expression vector. * for *p* < 0.05; ** for *p* < 0.01.

To elucidate the function of *ADAMTS1* in sheep follicular granulosa cells, four distinct siRNA sequences targeting *ADAMTS1* were designed and synthesized (Figure 2A). These siRNAs were transfected into granulosa cells, and after 24 h, the suppression of *ADAMTS1* expression was evaluated using quantitative real-time PCR and Western blot assays. Among the tested siRNAs, si-ADAMTS1-2 demonstrated the most effective knockdown efficiency relative to the control group (*p* < 0.05) (Figure 2B).

An overexpression construct for *ADAMTS1* was generated by PCR amplification of its coding sequence (CDS), resulting in a 2903 bp fragment (Figure 2C). The pcDNA3.1-EGFP vector was selected as the backbone and linearized by double digestion with EcoR I and BamH I, yielding a 6750 bp fragment corresponding to the empty vector (Figure 2D). The *ADAMTS1* CDS fragment was ligated into the linearized vector to create the EGFP-*ADAMTS1* overexpression plasmid, with a total size of 9657 bp. Verification of the recombinant plasmid through EcoR I and BamH I double digestion produced six distinct fragments measuring 6750 bp, 994 bp, 900 bp, 475 bp, 387 bp, and 151 bp (Figure 2D). Subsequent DNA sequencing confirmed that the inserted *ADAMTS1* sequence perfectly matched the reference ovine *ADAMTS1* gene (Figure 2G). These findings confirm the successful construction of the ovine *ADAMTS1* overexpression plasmid (Figure 2E). To assess transfection efficiency and expression, the EGFP-*ADAMTS1* plasmid was introduced into ovine ovarian granulosa cells. After 24 h, quantitative real-time PCR and Western blot analyses revealed that *ADAMTS1* expression was elevated by approximately 105-fold relative to the control group (*p* < 0.01) (Figure 2F).

### 3.3. ADAMTS1 Enhances the Proliferative Capacity of Ovarian Granulosa Cells

**Figure 3:**
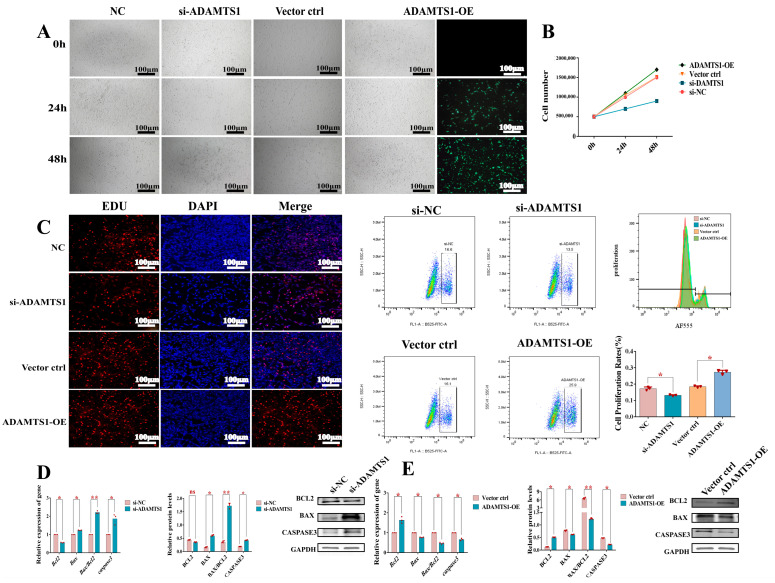
*ADAMTS1* enhances proliferation in follicular granulosa cells. (**A**) Morphological observations of granulosa cells at 0, 24, and 48 h post *ADAMTS1* knockdown and overexpression. *ADAMTS1* knockdown significantly reduced cell numbers, whereas *ADAMTS1* overexpression markedly increased cell numbers and enhanced EGFP fluorescence intensity in transfected ovarian granulosa cells. (**B**) Quantification of granulosa cell numbers at 0, 24, and 48 h following modulation of *ADAMTS1* expression. At 48 h post-transfection, *ADAMTS1* knockdown significantly reduced granulosa cell numbers by 40.0% (*p* < 0.01), while its overexpression enhanced cellular proliferation by 1.13-fold (*p* < 0.01). (**C**) Flow cytometric analysis of apoptosis in granulosa cells subjected to *ADAMTS1* silencing and overexpression. Relative to control groups, *ADAMTS1* knockdown reduced the proliferation rate of EdU-positive granulosa cells (GCs) to 76.3% of baseline levels (*p* < 0.01), whereas ADAMTS1 overexpression enhanced proliferative activity to 148.2% (*p* < 0.01). (**D**) After knockdown of *ADAMTS1*, the mRNA and protein expression levels of *Bcl2* (*p* < 0.05), *Bax* (*p* < 0.05), and *caspase3* (*p* < 0.05) in the granulosa cells were significantly increased. (**E**) After overexpression of *ADAMTS1*, the mRNA and protein expression levels of *Bcl2* (*p* < 0.05), *Bax* (*p* < 0.05), and *caspase3* (*p* < 0.05) in the granulosa cells were significantly decreased. ns for not significant; * for *p* < 0.05; ** for *p* < 0.01.

Granulosa cell (GC) proliferation is essential for proper ovarian follicle development, with disruptions in GC proliferation or apoptosis contributing to follicular abnormalities [24]. To elucidate the role of *ADAMTS1* in ovine granulosa cell proliferation, we manipulated *ADAMTS1* expression through targeted knockdown and overexpression at multiple time points (0, 24, and 48 h) and monitored changes in cell number. Our findings revealed that while control cells exhibited robust proliferation at 24 and 48 h, *ADAMTS1* silencing significantly decreased granulosa cell counts, whereas *ADAMTS1* overexpression substantially enhanced cell proliferation (Figure 3A). At 48 h post-transfection, *ADAMTS1* knockdown significantly reduced granulosa cell numbers by 40.0% (*p* < 0.01), while its overexpression enhanced cellular proliferation by 1.13-fold (*p* < 0.01) (Figure 3B). Moreover, proliferative activity was assessed via flow cytometry using EdU incorporation assays. The percentage of EdU-positive granulosa cells was markedly reduced following *ADAMTS1* knockdown but significantly elevated in cells overexpressing *ADAMTS1* compared to controls (Figure 3C).

To elucidate the underlying mechanisms by which *ADAMTS1* modulates granulosa cell proliferation, we analyzed the expression of apoptosis-related markers *Bcl2*, *Bax*, and *caspase3* in ovarian granulosa cells following *ADAMTS1* knockdown and overexpression using quantitative real-time PCR and Western blot analyses. *Bcl2* serves as a key anti-apoptotic factor, whereas *Bax* and *caspase3* are established pro-apoptotic mediators. Relative to the control group, *ADAMTS1* silencing resulted in a downregulation of *Bcl2* accompanied by marked upregulation of *Bax*, the *Bax*/*Bcl2* ratio (*p* < 0.01), and *caspase3* expression (*p* < 0.05) (Figure 3D). Conversely, *ADAMTS1* overexpression significantly elevated *Bcl2* levels (*p* < 0.05) while suppressing *Bax*, the *Bax*/*Bcl2* ratio (*p* < 0.05), and *caspase3* expression (*p* < 0.05) (Figure 3E). These findings suggest that *ADAMTS1* promotes granulosa cell proliferation, in part, by modulating apoptotic pathways.

### 3.4. Overview of RNA-Seq and Enrichment Analysis of DEGs

**Figure 4:**
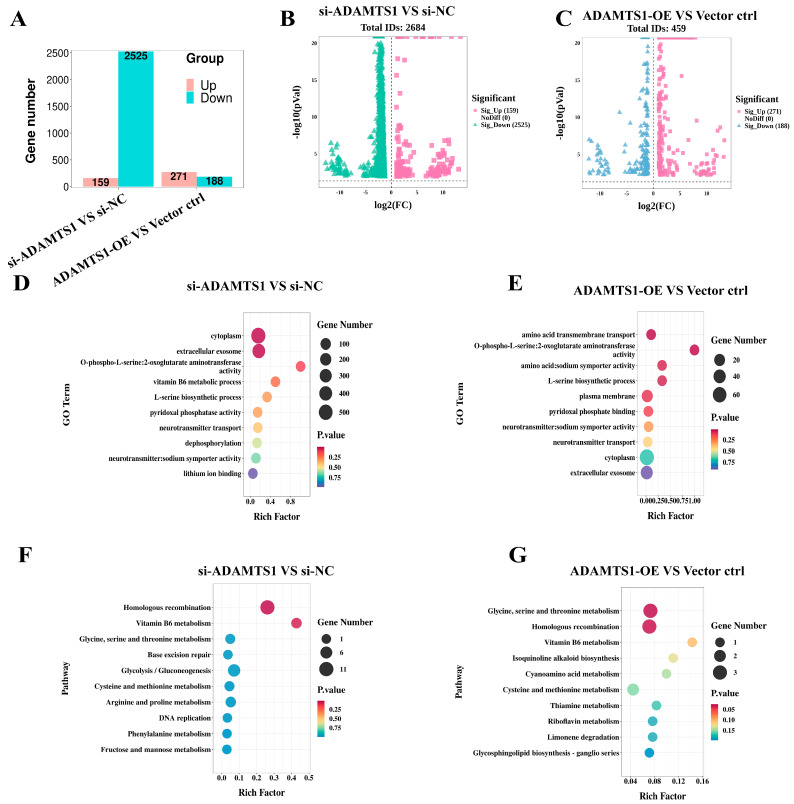
Summary of RNA sequencing results and functional enrichment analysis of differentially expressed genes (DEGs). (**A**) Quantification of upregulated and downregulated DEGs in *ADAMTS1* knockdown and overexpression groups. (**B**) Volcano plot illustrating DEGs identified between the *ADAMTS1* knockdown group and control. Transcriptomic analysis identified 2684 differentially expressed genes (DEGs), comprising 159 upregulated (5.9%) and 2,525 downregulated (94.1%) transcripts, based on the established cutoff criteria (*p* < 0.05, |log2 fold change| ≥ 1). (**C**) Volcano plot depicting DEGs between the *ADAMTS1* overexpression group and control. Transcriptomic analysis identified 459 differentially expressed genes (DEGs), comprising 271 upregulated (59.0%) and 188 downregulated (41.0%) transcripts, based on the established cutoff criteria (*p* < 0.05, |log2 fold change| ≥ 1). (**D**) Gene Ontology (GO) enrichment analysis for DEGs comparing the *ADAMTS1* knockdown group with control. (**E**) GO enrichment analysis for DEGs between the *ADAMTS1* overexpression group and control. (**F**) Kyoto Encyclopedia of Genes and Genomes (KEGG) pathway enrichment analysis for DEGs in the *ADAMTS1* knockdown versus control comparison. (**G**) KEGG pathway enrichment for DEGs in the *ADAMTS1* overexpression versus control comparison.

To investigate the molecular mechanisms regulated by *ADAMTS1* in granulosa cells, transcriptomic profiling was conducted following *ADAMTS1* knockdown and overexpression. High-quality RNA-seq data totaling 72.19 Gb were generated, with individual samples producing between 5.12 Gb and 7.03 Gb of clean reads. Quality metrics indicated Q30 base percentages ranging from 97.96% to 99.31% and an average GC content of 51.46%. Alignment to the reference genome yielded mapping rates between 91.40% and 97.72% across samples. Differential expression analysis, applying thresholds of adjusted *p*-value < 0.05 and |log2 fold change| ≥ 1 (Figure 4A), identified 2684 genes exhibiting significant expression changes in the ADAMTS1 knockdown group relative to controls, with 159 genes upregulated and 2525 downregulated (Figure 4B). Conversely, 459 differentially expressed genes were detected in the overexpression group compared to controls, including 271 upregulated and 188 downregulated genes (Figure 4C).

Gene Ontology (GO) and Kyoto Encyclopedia of Genes and Genomes (KEGG) enrichment analyses were conducted on the identified differentially expressed genes (DEGs), with emphasis placed on the top ten GO terms and KEGG pathways. In the GO analysis comparing the *ADAMTS1* knockdown group to the control, DEGs were predominantly enriched in the Cellular Component category, particularly within the cytoplasm and extracellular exosome. Molecular Function annotations highlighted enrichment in O-phospho-L-serine: 2-oxoglutarate aminotransferase activity and vitamin B6 metabolic processes. Furthermore, Biological Process categories implicated these genes in L-serine biosynthesis and pyridoxal phosphatase activity (Figure 4D). Similarly, in the *ADAMTS1* overexpression versus control comparison, DEGs showed significant enrichment in the cytoplasm and extracellular exosome (Cellular Component). Molecular Functions associated with these DEGs included O-phospho-L-serine: 2-oxoglutarate aminotransferase activity and amino acid: sodium symporter activity, while Biological Processes primarily involved amino acid transmembrane transport and L-serine biosynthesis (Figure 4E). Collectively, these findings demonstrate that the DEGs are chiefly involved in serine metabolism, localizing predominantly to cytoplasmic and extracellular exosomal compartments, and functionally linked to enzymatic activities critical for L-serine biosynthesis and related metabolic pathways.

KEGG pathway enrichment analysis revealed that, in comparison to the control, the differentially expressed genes (DEGs) in the *ADAMTS1* knockdown group were predominantly enriched in the following top ten pathways: homologous recombination, vitamin B6 metabolism, glycine, serine, and threonine metabolism, base excision repair, glycolysis/gluconeogenesis, cysteine and methionine metabolism, arginine and proline metabolism, DNA replication, phenylalanine metabolism, and fructose and mannose metabolism (Figure 4F). Conversely, DEGs identified in the *ADAMTS1* overexpression group relative to the control showed significant enrichment in glycine, serine, and threonine metabolism, homologous recombination, vitamin B6 metabolism, isoquinoline alkaloid biosynthesis, cyanoamino acid metabolism, cysteine and methionine metabolism, thiamine metabolism, limonene degradation, riboflavin metabolism, and glycosphingolipid biosynthesis—ganglio series (Figure 4G).

Comparative analysis of differentially expressed genes (DEGs) between the *ADAMTS1* knockdown and overexpression groups, each relative to their respective controls, revealed common enrichment in several key pathways. These include glycine, serine, and threonine metabolism, homologous recombination, vitamin B6 metabolism, as well as cysteine and methionine metabolism. Based on these findings, we propose that *ADAMTS1* may regulate the proliferation of ovarian granulosa cells by modulating these metabolic and DNA repair pathways, highlighting a potential mechanistic link between *ADAMTS1* activity and granulosa cell function.

### 3.5. Functional Analysis of DEGs

**Figure 5:**
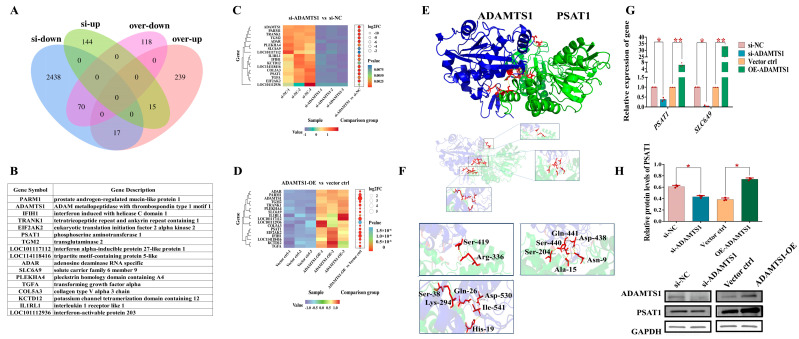
Functional characterization of DEGs. (**A**) Venn diagram illustrating the overlap of DEGs across four groups: downregulated and upregulated genes following *ADAMTS1* knockdown, and downregulated and upregulated genes following *ADAMTS1* overexpression. Through comparative analysis, it was found that in all comparison groups, there were 17 differentially expressed genes (*p* < 0.05, |log2 fold change| ≥ 1). (**B**) Detailed gene information for the 17 co-expressed DEGs (co-DEGs). (**C**) Heatmap depicting the expression patterns of the 17 co-DEGs in the *ADAMTS1* knockdown group. (**D**) Heatmap illustrating the expression profiles of the 17 co-DEGs in the *ADAMTS1* overexpression group. (**E**) Protein-protein molecular docking analysis between ADAMTS1 (colored blue) and PSAT1 (colored green). (**F**) Predicted interaction interface highlighting critical amino acid residues involved in binding: ADAMTS1 key residues include Ser419, Ser440, Gln441, Asp438, Lys294, Ile541, and Asp530; PSAT1 key residues include Arg336, Asn9, Ala15, His19, Gln26, and Ser38. (**G**) The mRNA expression levels of *PSAT1* and *SLC6A9* in the *ADAMTS1* knockdown group and the overexpression group. (**H**) The protein expression levels of PSAT1 in the *ADAMTS1* knockdown group and the overexpression group. * for *p* < 0.05; ** for *p* < 0.01.

We conducted Venn diagram analysis to identify overlapping differentially expressed genes (DEGs) among four groups: downregulated genes in the *ADAMTS1* knockdown group, upregulated genes in the *ADAMTS1* knockdown group, downregulated genes in the *ADAMTS1* overexpression group, and upregulated genes in the *ADAMTS1* overexpression group. This analysis revealed 17 shared DEGs between the downregulated genes in the knockdown group and the upregulated genes in the overexpression group (Figure 5A,B). Notably, *PSAT1* and *SLC6A9* were significantly downregulated following *ADAMTS1* knockdown and markedly upregulated upon *ADAMTS1* overexpression (Figure 5C,D).

Previous pathway enrichment analysis indicated that these common DEGs were predominantly involved in glycine, serine, and threonine metabolism as well as vitamin B6 metabolism pathways (Figure 4G). *PSAT1* functions as a critical rate-limiting enzyme within the glycine and serine biosynthetic pathway [16], playing a role in the synthesis of folate, methionine, and vitamin B6—cofactors essential for various biochemical processes [20]. Furthermore, *PSAT1* has been implicated in promoting cellular proliferation by modulating the cell cycle during the G2/M phase [25]. To elucidate the potential regulatory interaction between *ADAMTS1* and *PSAT1*, molecular docking analysis was performed, demonstrating a stable binding interface between the two proteins (Figure 5E,F).

We further verified the changes in the expression level of *PSAT1* through Q-PCR and WB. It was found that the expression levels of *PSAT1* mRNA and protein in the knockdown group were significantly decreased (*p* < 0.05), while those in the overexpression group were significantly upregulated (*p* < 0.05), which was consistent with the omics data (Figure 5G,H). These findings suggest that *ADAMTS1* may exert its influence on granulosa cell proliferation through direct interaction with *PSAT1*, highlighting a novel molecular mechanism warranting further investigation.

During the process of follicular development, multiple amino acid transporters exhibit active roles within both oocytes and granulosa cells, facilitating follicle maturation [26]. Our results demonstrate that silencing *ADAMTS1* leads to a significant reduction in the expression of the glycine transporter GLYT1 (*SLC6A9*), while *ADAMTS1* overexpression results in a marked increase in *SLC6A9* levels. We verified the changes in the expression level of *SLC6A9* through Q-PCR. It was found that the mRNA expression level of *SLC6A9* in the knockdown group was significantly decreased (*p* < 0.05), while that in the overexpression group was significantly upregulated (*p* < 0.01), which was consistent with the omics data (Figure 5G). Cumulus cells provide essential metabolites such as amino acids, pyruvate, and other intermediates to the oocyte through gap junctions [27], suggesting that these cells may function as a storage reservoir for glycine [28]. Therefore, we propose that *ADAMTS1* modulates amino acid metabolism in ovarian granulosa cells, potentially through the regulation of *SLC6A9* expression.

## 4. Discussion

Sheep represent an important indigenous livestock species in the Inner Mongolia region, where their reproductive efficiency plays a pivotal role in determining the economic productivity of pastoral farming systems. The reproductive capacity of livestock is inherently associated with the highly regulated and complex process of ovarian folliculogenesis [28], making detailed investigations into follicular development in sheep essential for improving breeding outcomes. Granulosa cells (GCs) are central to this process, as their proliferation and differentiation support follicular maturation and ovulation, while their apoptosis or degeneration leads to follicular atresia [29]. Therefore, the functional integrity and developmental dynamics of GCs are critical for ensuring normal follicular development. Elucidating the roles and regulatory mechanisms of GCss in the ovary offers key insights into the progression of folliculogenesis and has potential implications for enhancing reproductive performance in sheep. In current studies on sheep, *ADAMTS1* has been identified as a key gene affecting sheep fecundity [12]. The SNP polymorphisms of *ADAMTS1* are involved in regulating litter size in Hu sheep and Small Tail Han sheep [13], and high fecundity is associated with the production of a large number of mature follicles [15]. Numerous studies have shown that *ADAMTS1* is involved in the processes of ovulation and fertilization in mammals [11]. In *ADAMTS1*-deficient mice, ovarian morphology is abnormal, the number of ovulations is significantly reduced, and follicular atresia occurs [8,10]. Therefore, *ADAMTS1* plays a critical role in follicular development. Our study found that ADAMTS1 is primarily expressed in the granulosa cells of sheep follicles, indicating that investigating the role of *ADAMTS1* in sheep granulosa cells is essential for understanding follicular development.

Most follicles undergo atresia during development, and apoptosis of granulosa cells is the main cause of follicular atresia [6]. Therefore, the normal proliferation of granulosa cells is crucial for follicular development [7]. We found that knockdown of *ADAMTS1* in granulosa cells significantly reduced the number of EdU-positive cells and significantly upregulated the expression levels of *Bax* (*p* < 0.05), *Bax*/*Bcl2* (*p* < 0.01), and *caspase3* (*p* < 0.05). In contrast, overexpression of *ADAMTS1* in granulosa cells significantly increased the number of EdU-positive cells and significantly downregulated the expression levels of *Bax* (*p* < 0.05), *Bax*/*Bcl2* (*p* < 0.05), and *caspase3* (*p* < 0.05). The process of apoptosis is executed and regulated by the Bcl and caspase families. BCL2 and BAX, two key members of the Bcl family, are representative anti-apoptotic and pro-apoptotic proteins involved in the regulation of cell proliferation [30]. *Bcl2* is a typical anti-apoptotic gene, while *Bax* is a pro-apoptotic gene; the ratio of *Bax*/*Bcl2* can determine whether a cell undergoes apoptosis [31,32]. The caspase family plays a decisive role in whether granulosa cells undergo apoptosis [33], and *caspase3* regulates granulosa cell apoptosis through both the mitochondrial pathway and the death receptor pathway [34]. Therefore, we speculate that knockdown of *ADAMTS1* inhibits the proliferation of granulosa cells, while overexpression of *ADAMTS1* promotes their proliferation.

In mammalian ovaries, the dynamic balance between cell proliferation and apoptosis is crucial for maintaining the homeostasis of granulosa cells [35]. A specialized subpopulation of granulosa cells, known as cumulus cells (CCs), surrounds and connects with the oocyte through gap junctions, forming the cumulus-oocyte complex (COC) [36]. Thus, oocytes and cumulus cells are metabolically interdependent. CCs not only act as a biological barrier between oocytes and the external environment but also allow the transfer of regulatory factors and metabolic substrates to the oocyte [37]. During oocyte maturation, amino acid transport by cumulus cells is essential [38]. In the absence of surrounding cumulus cells, oocytes exhibit metabolic disorders, which significantly affect fertilization and embryonic development in cattle, pigs, and mice [39]. Supplementing in vitro oocyte culture media with essential and non-essential amino acids significantly increases maternal mRNA levels in oocytes and promotes embryonic development [40]. Therefore, the amino acid metabolism between granulosa cells and oocytes plays a key role in oocyte and follicular development [5]. We found that knocking down *ADAMTS1* inhibited, while overexpressing *ADAMTS1* promoted, the glycine, serine, and threonine metabolism and vitamin B6 metabolism pathways. Among them, the glycine, serine, and threonine metabolism pathway is a central network in amino acid metabolism, providing cells with substances such as glycine and serine. Vitamin B6 serves as a coenzyme and is involved in the biosynthesis and degradation of amino acids [19]. Moreover, our results showed that *PSAT1* was significantly enriched in both the glycine, serine, and threonine metabolism and vitamin B6 metabolism pathways. PSAT1 is a rate-limiting enzyme in the glycine, serine and threonine metabolism pathway, which catalyzes the conversion of 3-phosphohydroxypyruvate to L-phosphoserine [16], and its catalytic activity is highly dependent on its cofactor. In current studies, *PSAT1* has been identified as an oncogene associated with metastasis and poor prognosis in various malignancies, including lung cancer, liver cancer, colorectal cancer, breast cancer, ovarian cancer, and endometrial cancer [6,22,23]. Studies have shown that *PSAT1* is involved in regulating cell proliferation in vitro; overexpression of *PSAT1* in human U937 cells significantly promotes cell proliferation [17], while knockdown of *PSAT1* significantly inhibits the cell cycle and promotes apoptosis in ovarian cancer cells [16]. In our transcriptome sequencing results, *PSAT1* expression was significantly downregulated after *ADAMTS1* knockdown and significantly upregulated after *ADAMTS1* overexpression, consistent with RT-PCR and Western blot validations. We hypothesize that *ADAMTS1* may regulate granulosa cell proliferation through *PSAT1*. To investigate the relationship between *ADAMTS1* and *PSAT1*, we performed protein-protein docking and found a stable binding interface between ADAMTS1 and PSAT1. Therefore, we speculate that *ADAMTS1* may affect granulosa cell proliferation by regulating the expression of *PSAT1* within the glycine, serine, and threonine metabolism and vitamin B6 metabolism pathways.

During follicular development, various amino acid transporters are active in both oocytes and granulosa cells, participating in the amino acid metabolic processes of the follicle [25]. Studies have shown that transporters in mouse granulosa cells (GCs) can mediate the uptake of L-serine into the cumulus-oocyte complex (COC), which is subsequently transferred to the growing and maturing enclosed oocytes [41], thereby supporting oocyte growth and maturation through amino acid metabolism [40]. We found that knockdown of *ADAMTS1* significantly downregulated the expression of the glycine transporter GLYT1 (*SLC6A9*), while overexpression of *ADAMTS1* significantly upregulated *SLC6A9* expression. We speculate that *ADAMTS1* may act as an upstream regulator of SLC6A9, thereby modulating amino acid metabolism. *SLC6A9* belongs to the classical glycine transport system (GLY) and is quiescent at the germinal vesicle (GV) stage but becomes activated during ovulation. GCs provide oocytes with amino acids, pyruvate, and intermediate metabolites via gap junctions [26], and thus cumulus cells may serve as a reservoir for glycine [27]. We speculate that *ADAMTS1* may regulate amino acid metabolism in ovarian granulosa cells by modulating the expression of SLC6A9, which in turn affects *PSAT1* in the glycine, serine, and threonine metabolism and vitamin B6 metabolism pathways, thereby influencing granulosa cell proliferation.

Our study predicts the upstream and downstream regulatory relationships among *ADAMTS1*, *PSAT1*, and *SLC6A9*, as well as potential binding sites between the ADAMTS1 and PSAT1 proteins. However, we have not yet directly demonstrated the interaction or confirmed whether ADAMTS1 directly regulates PSAT1. Therefore, our future work will focus on investigating the precise molecular regulatory mechanisms between *ADAMTS1* and *PSAT1* at the genetic, transcriptional, post-transcriptional, and translational levels. Normal proliferation of granulosa cells is crucial for follicular development, and the reproductive performance of livestock is closely linked to the complex process of follicle development. Through our research, we aim to elucidate the mechanisms by which *ADAMTS1* affects granulosa cell proliferation and hope to provide a preliminary theoretical foundation for reproductive studies in sheep.

## 5. Conclusions

Therefore, we speculate that *ADAMTS1* may participate in amino acid metabolism by regulating the transporter protein SLC6A9, which in turn modulates the expression of *PSAT1* in the glycine, serine, and threonine metabolism and vitamin B6 metabolism pathways, ultimately influencing granulosa cell proliferation. However, our study has not directly demonstrated the interaction between ADAMTS1 and PSAT1 proteins, nor confirmed whether this regulation is direct. Thus, we will continue to investigate the precise molecular regulatory mechanisms between *ADAMTS1* and *PSAT1*. Through our research, we aim to elucidate the molecular mechanisms by which *ADAMTS1* affects granulosa cell proliferation, providing a preliminary theoretical foundation for reproductive studies in sheep.

## Figures and Tables

**Table 1:** Primers for amplifying the ADAMTS1 gene.

Gene Name		Forward and Reverse Primer Sequences (5′→3′)
*ADAMTS1*	F	aaGCstgtgaccgGCsGCsctacgaattcGCsCACCatGCsaGCsGCsGCsggt
	R	CCccATCGATggACCGGTcgGGATCCactGCsactctGCscattgtGCs

**Table 2:** Real-time quantitative PCR primer sequences.

Gene Name		Forward and Reverse Primer Sequences (5′→3′)	Annealing Temperature/°C
*ADAMTS1*	F	CCCCATGTAGCsCCAGATTCC	58 °C
	R	ATCATGGTAGCsCGGGTCTTG	
*Bcl2*	F	GGGGTCATGTGTGTGGAGAG	58 °C
	R	TGCsAGCsTCCACAAAGGCsGTC	
*Bax*	F	TTCCGACGGCsAACTTCAACT	58 °C
	R	CTGATCAACTCGGGCsACCTT	
*caspase3*	F	TTCAGAGGGGACTGTTGCsAG	58 °C
	R	CAGTCCAGTTCTGTGCsCTCG	
*GAPDH*	F	GTTTGTGATGGGCsGTGAACC	58 °C
	R	GCsGTGGACAGTGGTCATAAGT	
*PSAT1*	F	AACCTTGTACGGGAATTGTT	58 °C
	R	CTCGGATCTGGAATTTTCGT	
*SLC6A9*	F	GCsTGCsAGTATGCsTCTGG	58 °C
	R	GAAGTAGGGGAACATGAAGG	

## Data Availability

The data presented in this study are available upon request from the corresponding author.

## Abbreviations

The following abbreviations are used in this manuscript:

ADAMTS1	A Disintegrin and Metalloproteinase with Thrombospondin Motifs 1
GCs	Granulosa Cells
OC	Oocyte
COC	Cumulus-Oocyte Complex
FSHR	Follicle-Stimulating Hormone Receptor
Bcl2	B-cell Lymphoma 2
Bax	BCL2-Associated X Protein
caspase3	Cysteine-Aspartic Acid Protease 3
GAPDH	Glyceraldehyde-3-Phosphate Dehydrogenase
PSAT1	Phosphoserine Aminotransferase 1
SLC6A9	Solute Carrier Family 6 Member 9
GO	Gene Ontology
KEGG	Kyoto Encyclopedia of Genes and Genomes
BP	Biological Process
CC	Cellular Component
MF	Molecular Function
RT-PCR	Reverse Transcription Polymerase Chain Reaction
PCR	Polymerase Chain Reaction
ECM	Extracellular Matrix
WB	Western Blot
DAB	3,3’-Diaminobenzidine
CDS	Coding DNA Sequence
DEGs	Differentially Expressed Genes
FDR	False Discovery Rate
HE	Hematoxylin and Eosin
